# Research advances on the hard seededness trait of soybean and the underlying regulatory mechanisms

**DOI:** 10.3389/fpls.2024.1419962

**Published:** 2024-06-26

**Authors:** Yongwang Sun, Yujie Gong

**Affiliations:** School of Agricultural Science and Engineering, Liaocheng University, Liaocheng, China

**Keywords:** soybean, hard seededness, seed coat structure, chemical component, QTL, gene

## Abstract

Soybean is one of the world’s most economically significant crops and is widely utilized as an essential source of vegetable protein and edible oil. Cultivated soybean is domesticated from its annual counterpart, wild soybean, which is considered valuable germplasm for soybean breeding. However, wild soybean accessions generally produce seeds with impermeable coats, a trait known as hard seededness (HS), which is beneficial for long-term seed survival but is undesirable for the uniform water absorption and germination of seeds, thus limiting the utilization of wild soybeans in breeding. In addition, moderate HS can isolate the embryo from the surrounding environment and is thus beneficial for long-term seed storage and germplasm preservation. The HS trait is primarily associated with the structure and chemical composition of the seed coat. Moreover, its development is also influenced by various environmental conditions, such as water and temperature. Genetic analysis has revealed that HS of soybean is a complex quantitative trait controlled by multiple genes or minor quantitative trait loci (QTL), with many QTLs and several causal genes currently identified. Investigating the physiological and molecular mechanisms underlying this trait is crucial for soybean breeding, production, and food processing. For this article, the literature was reviewed and condensed to create a well-rounded picture of the current understanding of internal and external factors, QTLs, causal genes, and the regulatory mechanisms related to the HS of soybean, with the aim of providing reference for future research and utilization of this trait.

## Introduction

Soybean [*Glycine max* (L.) Merr.] is an important leguminous crop due to its high protein and oil contents. Generally, oil, protein, and carbohydrate account for approximately 20%, 40%, and 35%, respectively, of the dry mass of cultivated soybean seeds, making it an excellent source of vegetable oil and protein for humans and livestock (Medic et al., 2014; Hudson, 2022). Cultivated soybean was domesticated from the annual wild soybean (*Glycine soja* Sieb. and Zucc.) ~5000 years ago, in the eastern half of north China and disseminated to Europe and America in the 18^th^ century (Li et al., 2008; Kim et al., 2012). Wild soybeans are distributed throughout East Asia, including most of China, Korea, Japan, and part of Russia, and have extremely rich genetic resources (Larson et al., 2014). Compared to their wild relatives, cultivated soybeans have undergone significant changes in growth habits, plant architecture, seed morphology, and composition under long-term targeted selection and improvement (Liu et al., 2007; Wen et al., 2009; Yuan et al., 2022). Cultivated soybeans display a bush-type growth habit with a stout primary stem and sparse branches, bearing large seeds that are generally prone to water absorption and germination. In contrast, wild soybeans produce a procumbent or climbing vine with slender, many-branched stems bearing small, coarse seeds that are impermeable to water (Zhou et al., 2010; Liu et al., 2021). During selective domestication and specific breeding improvement, approximately 50% of the genetic diversity and 81% of rare alleles in wild soybeans have been lost (Hyten et al., 2006). At present, most soybean cultivars worldwide face a “genetic bottleneck” (Guo et al., 2010). Many of the soybean cultivars come from a few ancestors (Li et al., 2008; Wysmierski and Vello, 2013; Li et al., 2023), and the narrow genetic basis and limited gene sources not only inhibit cultivar improvement, but also increase their vulnerability to abiotic stresses, disease, and pests (Zhou et al., 2015).

Due to not being influenced by human selection, wild soybeans contain abundant genetic resources, which have advantages such as multiple flowers and pods, high protein content, high reproductive coefficients, and tolerance to infertility (Liu et al., 2007; Zeng et al., 2012). In addition, wild and cultivated soybeans exhibit normal pairing of meiotic chromosomes and hybridize easily (Liu et al., 2007; Kim et al., 2012). Thus, wild soybeans are an extraordinarily important gene pool for soybean breeding, which can enrich and broaden the genetic background of cultivated soybeans, and provide beneficial traits and genetic resources (Chen et al., 2017; Kim et al., 2021; Aleem et al., 2022). As reviewed by Zhuang et al. (2022), wild soybeans had been used as genetic resource to improve the biotic and abiotic resistance and yield-related traits of soybean cultivars. However, seeds from wild soybean accessions generally exhibit a hard seededness (HS) trait, which impedes the seeds’ water absorption even after long periods of soaking until external facors (e.g. fluctuating temperatures, fire, drying, freezing) render the seed coat permeable to water (Baskin et al., 2000). The HS trait, which has been actively selected against by plant breeders since uniform and rapid emergence is impeded, may appear as a “linkage drag” in hybrid breeding programs, seriously limiting the utilization of wild soybean resources (Sharma et al., 2020). For example, the HS rate of the F_2_ and F_3_ seeds obtained by crossing the *G. max* line T14R1251–70 and *G. soja* accession LNTL was 89.5% and 96.5%, respectively (Liang et al., 2023). This will undoubtedly increase the difficulty of the subsequent breeding process. By contrast, moderately impermeable seed coats can prevent seed decay and prolong seed lifespan by isolating the embryo from the surrounding environment and is thus beneficial for prolonging the storage time of seeds and germplasm preservation, especially in humid environments (Hartwig and Potts, 1987; Kulik and Yaklich, 1991; Roy et al., 1995; Tyler, 1997; Moore and Yaklich, 2000). Therefore, breeding programs have been aimed at developing soybean cultivars with seed coats that are reasonably permeable and fairly strong (Kilen and Hartwig, 1978; Moore and Yaklich, 2000; Ma et al., 2004). For example, soybean breeding lines exhibiting HS trait, such as D67–5677, D67–5679, D86–4565, and D87–4647, had been most used in breeding cultivars that are well adapted to the southern U.S.A (Yaklich and Cregan, 1989; Moore and Yaklich, 2000). Understanding the physiological and molecular mechanisms controlling the HS trait of soybean is of great significance for its application. Previous studies have extensively investigated the seed coat structure and components, environmental factors, quantitative trait loci (QTL), causal genes, and regulatory mechanisms associated with HS. This paper aims to provide an overview of the research advances related to the HS trait of soybean and propose future research directions in this field.

### Definition and implications of the HS trait

HS, also known as physical dormancy, seed coat-imposed dormancy, or “stone” or “impermeable” seeds, has been found in ~15 angiosperm families, of which the Leguminosae is the largest (Meyer et al., 2007; Baskin and Baskin, 2014; Paulsen et al., 2014; Wen et al., 2024). In contrast, seeds that are prone to water absorption are termed “soft” or “permeable” seeds. HS is the second most common type of seed dormancy after physiological dormancy and occurs when the seed coat becomes thick or hard, impermeable to surrounding water and air, and keeps the embryo in a viable state for a long time (Foley, 2001; Finch-Savage and Leubner-Metzger, 2006; Paulsen et al., 2013). HS is a biologically beneficial trait that plants have acquired over a long evolutionary process to adapt to environmental changes. For example, seeds with this trait are not easily detected by rodents through their sense of smell, are difficult to digest, and can spread to a larger region as the forager migrates (Nik and Parbery, 1977). After encountering harsh environments such as floods and fires, many of the hard seeds remain viable and can quickly resettle on areas, as long as the environmental conditions are suitable for seed germination (Ferrandis et al., 1999). Hard seeds may benefit the continuation of species in nature by preserving a seed stock for many years so that seeds can germinate at different times to avoid sudden disasters (Li and Huang, 1994; Mohamed-Yasseen et al., 1994; Koornneef et al., 2002). The hard seed coat isolates the embryo from the outside air and water under rainy conditions to maintain the long-term vitality of the seed and avoid pathogen invasion (Dalling et al., 2011). For example, some legume seeds have remained viable for more than 100 years (Rolston, 1978).

Like other plants, hard seeds from wild soybean accessions have higher vitality and can survive for at least five to seven years in the soil seed bank, which is beneficial for adaptation to unstable and unpredictable environmental conditions to ensure population continuity (Li and Huang, 1994; Heatherly et al., 1995). The longevity of wild soybeans can be attributed to their combined physical and physiological dormancy (Sun et al., 2015; Wang et al., 2018). In contrast, most soybean landraces and cultivars can imbibe water in a short time without scarification of the seed coat, although considerable variation exists in their degree of seed coat permeability (Chachalis and Smith, 2000; Sakamoto et al., 2004; Kebede et al., 2014). For example, hard seed ratio varied by an average of 15% for eleven strains in Mississippi (Hartwig and Potts, 1987). Mullin and Xu (2001) reported that three of the six tested soybean cultivars from U.S.A., Canada and Japan produce hard seeds with varying degrees (e.g. Bobcat, 3.1%; Harosoy, 5.6%; OX 951, 72.4%), while the others all produce non-hard seeds. Guo et al. (2002) reported that five of the seven test soybean cultivars from northeast China produce a relatively small proportion of hard seed (HS ratio ranging from 0.5% - 3.0%). Moreover, adverse climatic conditions such as drought and heat during the growing season, especially as the seed matures, may increase the ratio of hard seeds in soybean production (Moore and Yaklich, 2000; Mullin and Xu, 2001). Currently, HS is often a disadvantageous trait for cultivated soybeans and seriously impedes seed production in agriculture (Meyer et al., 2007). Sowing of hard soybean seeds leads to a dramatic decrease in field emergence rate and uniformity, resulting in different seedling density and mass weed growth, which seriously decreases the sowing quality and ultimately reduces the soybean yield (Potts et al., 1978; Tyler, 1997). Under the continuous cropping system, if hard seeds germinate the following year, they will mix with subsequent soybean seeds (sometimes from different cultivars), and the harvest quality will be decreased (Keith and Delouche, 1999; Meyer et al., 2007; Abbo et al., 2008). In the food industry, rapid and uniform hydration of soybean seeds is necessary for subsequent seed processing. Hard seeds will not only affect the sensory quality of whole-soy foods, including soybean sprouts, natto, and baked beans, but also reduce the milling quality of crack-soy foods such as soy milk, soy sauce, soy paste, miso, and tofu (Zhang et al., 2008; Smýkal et al., 2014). To ensure uniform water absorption and germination of soybean seeds with the HS trait, multiple methods, such as mechanical scratching, temperature upheaval, or soaking seeds with strong acid or alkaline solutions, have to be employed to break their seed coat (Argel and Paton, 1999; Moore and Yaklich, 2000; Shao et al., 2007; Sun et al., 2014). Undoubtedly, these steps increase the processing costs and may also decrease the seed vitality.

In addition to the adverse effects on agricultural production and food processing, the HS trait has also shown valuable utilization in certain agricultural practices. For example, if adverse weather (e.g. humid conditions or rainy season) occurs before seed maturity or harvest, the hard seed coat can hinder the diffusion of water and air to the embryo to prevent seed spoilage. Different with wild soybean, cultivated soybean seeds generally lack physiological dormancy and are prone to deterioration in seed viability under prolonged storage (Qutob et al., 2008; Ramakrishna et al., 2018; Wang et al., 2018). Thus, a moderate or rational level of HS can delay the embryo’s water absorption from the surrounding environment, resist pathogen invasion, and help the seed maintain strong vitality and good quality for a longer period (Potts et al., 1978; Keim et al., 1990; Kulik and Yaklich, 1991; Roy et al., 1995; Wang, 1999). In addition, HS may be associated with high calcium content in the seed coat and can therefore potentially enhance the nutritional value of soy-based foods (Saio et al., 1973; Saio, 1976; Chen et al., 2001; Zhang et al., 2009; Sun et al., 2015). Therefore, a deep understanding of the physiological and molecular basis of the HS trait is necessary for the rational application of wild soybean resources and the HS trait in soybean cultivar improvement, soybean production, and soy food processing practices.

## Internal and external factors affecting the HS trait

HS in soybean is genetically and environmentally determined. In terms of soybean seed characteristics, HS is mainly related to the structure and chemical composition of the seed coat (Ma et al., 2004; Meyer et al., 2007; Qutob et al., 2008), although several studies have emphasized the role of the hilum or micropyle in water absorption (Hyde, 1954; McDonald et al., 1988; Pietrzak et al., 2002; Xu et al., 2009). The seed coat, the outer covering of the seed, is largely derived from the integuments of the maternal tissue after fertilization, consisting of an embryo and nutritive tissue (Boesewinkel and Bouman, 1995). It can not only transfer nutrients from the mother plant to the embryo during seed development but also protects the embryo from mechanical damage or pathogen invasion (Souza and Marcos-Filho, 2001; Brooker et al., 2007). Meyer et al. (2007) suggested that the imbibition process by soybean seeds occurs in two distinct phases, the first dominated by hydration of the seed coat and the second by hydration of the cotyledons, which is rate-limited by the former. After removal of the seed coats, no significant difference was found in the water absorption rates between permeable and impermeable seeds. The water permeability rate of the hard seed coat was found to be five times lower than that of the permeable seed, indicating that seed coat permeability is the main factor leading to the HS trait. Moreover, permeable seeds typically imbibed water initially through their dorsal sides, opposite the hilum, forming wrinkles in their seed coats and delivering water to the underlying cotyledons (Meyer et al., 2007). The soybean seed coats are covered by a cuticle, which is subtended by closely packed palisade macrosclereids (also known as Malpighian layer cells), thick-walled osteosclereids (also known as hourglass cells), crushed parenchyma, and an intact aleurone layer (Yaklich et al., 1992; Shao et al., 2007). The cuticle is a structural component of the seed coat that provides a potential barrier to water movement (Heredia, 2003). Using scanning electron microscopy (SEM), Ma et al. (2004) observed the surface structure of seed coats from six soybean cultivars exhibiting different water imbibition rates. They found that the cuticle of permeable seeds has many microscopic cracks that typically penetrate the outer periclinal walls of the palisade layer, which occur more frequently on the dorsal side than in other regions. In contrast, the cuticle of hard seeds (*G.max* cv. OX951, from U.S.A.) is relatively intact on the whole of the seed coat surface. Moreover, isolated pieces of cuticle from hard seeds were found to be stronger and less prone to breaking when handled than those isolated from permeable seeds. After immersion in water, the typical water absorption phenomenon of seed coat wrinkles are first observed on the dorsal side, and then the wrinkles extend to the lateral side. In contrast, a fluorescent tracer dye assay showed that the hilum, micropyle, and raphe were not sites of initial water entry. Therefore, the authors suggested that the integrity of the cuticle and its underlying palisade layer at the dorsal site is the key factor that determines the permeability of a soybean seed coat (Ma et al., 2004). A time-course observation of the seed coat from the developing seeds of different soybean cultivars showed that at the L2b stage, when seed expansion was maximal, cracks in the cuticle start to appear on the dorsal region of permeable seeds, but not impermeable seeds, regardless of their genotype (Ranathunge et al., 2010). These results further confirm that it is the outermost cuticle, especially when located on the dorsal side of seeds, that controls water flow into soybean seeds during imbibition. In contrast, the determining factors of seed coat permeability in wild soybeans may differ from those in cultivated soybean seeds exhibiting HS trait. The micropyle of wild soybean seeds cannot be observed under SEM due to them being covered by the cuticle. However, after treatment with concentrated sulfuric acid, which leads to the shedding of the cuticle, the micropyle of wild soybean seeds is exposed and the seeds become permeable (Xu et al., 2009). Thus, Xu et al. (2009) believed that the micropyle may be the main channel for water uptake. Additional experiments, such as the fluorescent tracer dye assay conducted by Ma et al. (2004), were needed to determine the initial site of water entry in wild soybeans.

In addition to the seed coat structures, the presence of certain chemicals on the seed coat are also related to the HS trait. Mullin and Xu (2001) determined the chemical components of the seed coats of six soybean cultivars, and found that there was no correlation between the concentration of any of the cations and the occurrence of HS, although many other studies have shown that there is a close relationship between the calcium content of the seed coat and the HS trait of cultivated soybean (Saio et al., 1973; Saio, 1976; Chen et al., 2001; Zhang et al., 2009; Sun et al., 2015). Interestingly, low pectin content and high hemicelluloses, particularly xylans, can reduce hydrophilicity and may lead to the occurrence of HS (Mullin and Xu, 2001). Shao et al. (2007) found that the outermost cuticle of hard seed coats contained a high amount of hydroxylated fatty acids relative to that of permeable seeds. Moreover, boiling hard wild soybean seeds in 1 M NaOH for 5 min released the ω-hydroxy fatty acid component of the cuticle and created holes in the seed coat surface, causing the seeds to become permeable. Three major phenolics, namely epicatechin, cyanidin-3-O-glucoside, and delphinidin-3-O-glucoside, were isolated from wild soybean seed coats, and the changes in epicatechin content were significantly positively correlated with the HS trait under different water conditions during seed development and under different gas conditions during seed storage (Zhou et al., 2010). Similarly, Vijayan et al. (2023) measured the phenols, tannins, proteins, trace elements, and metabolites in the seed coats of two soybean cultivars exhibiting different levels of water permeability. They found that phenols are the main compounds responsible for seed coat impermeability, which may provide strength to the seed coat.

Previous studies have also shown that the HS trait of soybean may also be related to seed color and size and is influenced by multiple environmental factors. Inheritance studies have found that there may be an association between pigmentation loci and HS in soybean, where dark-colored seeds determined by genetic factors generally exhibit lower imbibition rates than light-colored seeds (Kuo, 1989; Sakamoto et al., 2004; Watanabe et al., 2004; Liu et al., 2007). However, several other studies have shown that seed coat pigmentation is not closely linked with HS, since many soybean lines without coloring possess an impermeable seed coat (Keim et al., 1990; Chachalis and Smith, 2000; Ma et al., 2004). Qutob et al. (2008) suggested a possible pleiotropic effect between pigmentation loci and permeability through their activity toward polyphenolic substrates that become impregnated in the cell wall. Small seeds, regardless of genotype, are found to be positively associated with HS of soybean (Calero et al., 1981; Nooden et al., 1985; Yaklich et al., 1986; Hill et al., 1986a; Ragus, 1987; Taira, 1990; Moore, 1991; Huang et al., 1999). Adverse growing conditions such as drought, low moisture, and high temperature, especially at the seeds’ late developmental stage, typically increase the occurrence of hard seeds in soybean; therefore, the hard seed ratio in cultivated soybean often vary between different years and geographic locations (Baciu-Miclaus, 1970; Hill et al., 1986b; Vieira et al., 1992; Argel and Paton, 1999; Egli et al., 2005; Kebede et al., 2014; Jaganathan and Harrison, 2024). As observed by Ranathunge et al. (2010), the cuticular cracks that are critical for seed permeability were induced by the internal hydrostatic forces of cotyledons when seed expansion was maximal. The reason why seeds with small sizes and those that encounter adverse climates are typically impermeable is probably because their cotyledons are unable to continue to expand rapidly later in development and are not prone to result in cuticular cracking.

## QTL mapping of HS trait in soybean

The localization and cloning of genes or QTLs for important traits are of great significance in crop breeding. In the past few decades, researchers have used many F_2_ populations and/or recombinant inbred lines (RILs) to perform extensive linkage analysis of QTLs associated with the HS trait of soybean. In general, researchers have found that the HS trait is a dominant trait in soybean, which is controlled by multiple genes or minor QTLs, although several papers have reported the monogenetic control of this trait (Kebede et al., 2014; Sun et al., 2015). Initially, Kilen and Hartwig (1978) studied the inheritance of HS in soybeans by crossing two soybean breeding lines, ‘Tracy’ and ‘D67–5679’ (producing 88% hard seed) from U.S.A., with different levels of seed coat permeability, and they speculated that as few as three major genes may control the variation in soybean HS. Using a F_2_ segregating population derived from a cross between *G. max* and *G. soja*, Keim et al. (1990) identified five restriction fragment length polymorphism markers, located on chromosomes 2, 3, 8, and 19, that were associated with variations in the HS trait. These markers and their epistatic interactions accounted for a total of 71% of variation in HS. Using a similar strategy, Sakamoto et al. (2004) found that at least two major QTLs and one minor QTL were involved in controlling the HS trait. Among them, a QTL near Satt459, a simple sequence repeat (SSR) marker on chromosome 2, could explain 23.8% and 38.5% of the phenotypic variations in the F_2_ and F_3_ generations, respectively. Using an F_2_ population derived from two soybean cultivars (non-HS cv. JS 71–05 and HS cv. Brisa soya-1 from India) exhibiting different levels of seed coat permeability, Singh et al. (2008) found that four SSR markers, located on chromosomes 6, 8, 12, and 15, were significantly correlated with seed coat permeability, explaining 3.9%–4.5% of the total phenotypic variation. Among these markers, Satt281 also showed significant association with electrolyte leaching and explained 5.6% of the total variation for this trait, which was negatively associated with seed vigor during germination. Based on two F_2_ populations derived from a cross and reciprocal cross made between two *G. max* accessions (non-HS cv. PI 587982A and HS cv. PI 594619 from U.S.A) exhibiting different levels of seed coat permeability, Kebede et al. (2014) found that the main locus controlling soybean seed coat hardness in *G. max* accession PI594619 is located in a 0.18-Mb interval between the markers Satt202 and Satt459 on chromosome 2.

**Figure 1:**
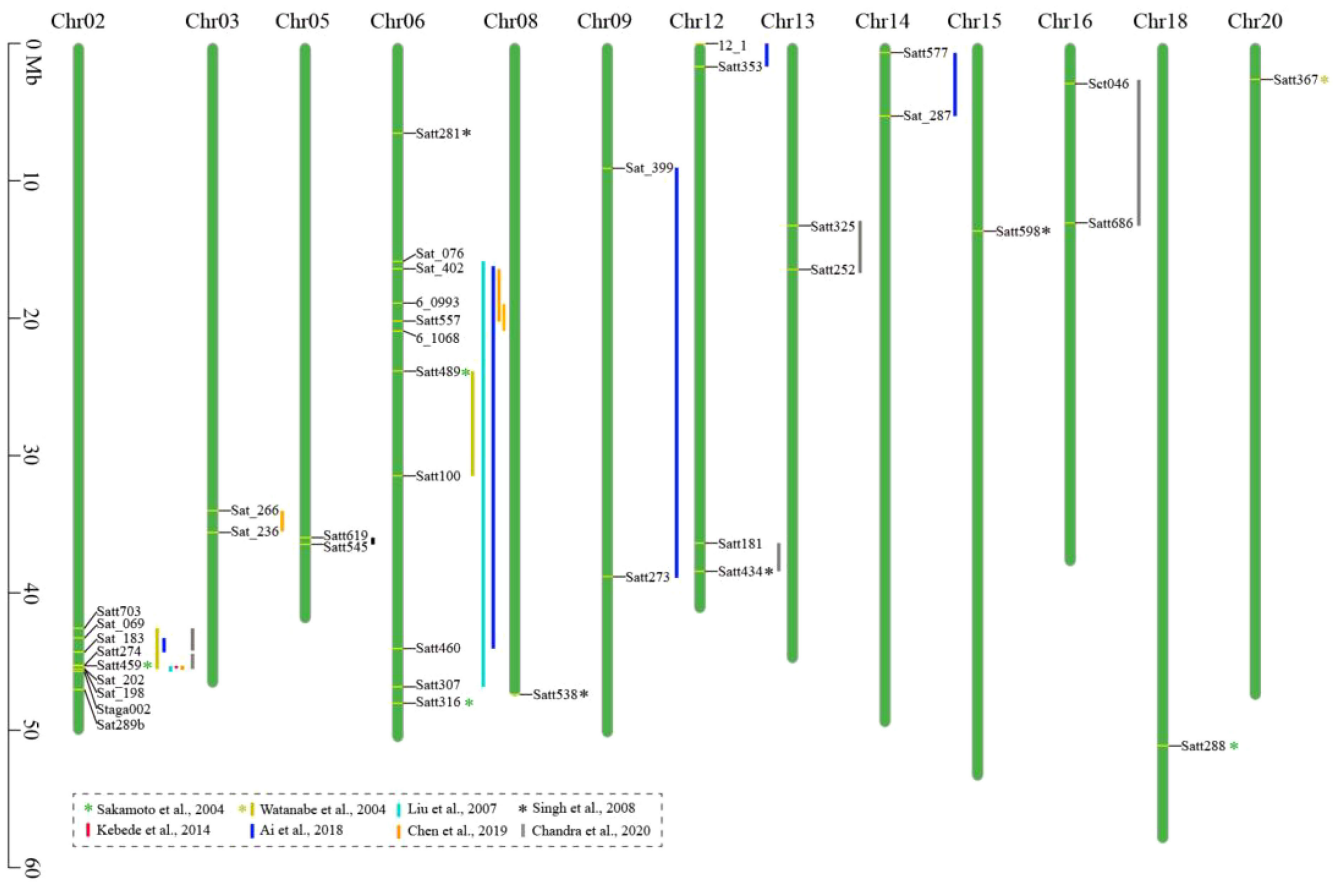
Chromosomal locations of the hard seededness (HS) quantitative trait loci (QTLs) of soybean.

Compared to the F_2_ segregating population, the advantage of using a RIL population is that replicated experiments at different locations and/or times can be carried out and enable researchers to evaluate the environmental effects. Based on a RIL population derived from a cross between *G.max* and semi-wild *G. max* lines, Watanabe et al. (2004) identified three QTLs (*RAS1–3*) on chromosomes 2, 6, and 20, which accounted for 10.6%, 26.0%, and 6.1% of the phenotypic variance of HS, respectively. Using a RIL population developed from a cross between *G. max* and *G. soja*, Liu et al. (2007) found two HS QTLs on chromosomes 2 and 6, which were consistently detected over 2 years, accounting for more than 42% and 13% of the phenotypic variations, respectively. Based on a RIL population developed from a cross between a permeable soybean cultivar and an impermeable soybean landrace, Ai et al. (2018) identified three QTLs for HS on chromosomes 2, 6, and 14, respectively, with a genetic contribution rate of 5.54%–12.94%. Additionally, four pairs of epistatic interaction QTLs were detected on chromosomes 2, 6, 9, 12, and 14, respectively, which explained 2.53%–3.47% of the phenotypic variation. Their results indicated that the epistatic interaction effect may play an important role in the genetic basis of the HS trait of soybean. Chen et al. (2019) constructed F_2_ and RIL populations by crossing a soybean cultivar with a wild soybean accession. Based on bulked segregant analysis and traditional linkage mapping, three HS QTLs were detected on chromosomes 2, 3, and 6, respectively, which explained 4.9%–23.3% of the phenotypic variation. Chandra et al. (2020) constructed 204 RILs by crossing two wild soybean accessions with a permeable soybean cultivar, and seven HS QTLs were mapped on chromosomes 2, 5, 12, 13, and 16, respectively, explaining 5.96%–39.67% of the phenotypic variations. Two main and stable QTLs distributed in tandem on chromosome 2 were consistent over the 3 years of testing, and they jointly explained 43.09%–62.92% of the phenotypic variations of the HS trait. The QTLs mapped and the linked markers identified in these studies are summarized in **Table 1** and **Figure 1**, which would be useful in breeding soybean cultivars with rational seed coat permeability by suitable genetic modifications.

**Table 1:** List of QTLs related to the HS trait of soybean.

Population information^#^	Chromosome	QTL	Marker(s)	Location	LOD	PVE(%)	Reference
Type: F_2_ P_1_: Tokei 780, *G. max*, Japan P_2_: **B01167**, *G. soja*, Japan	6	NG	Satt316	48016322	NG	24.8	Sakamoto et al., 2004
	6	NG	Satt489	23848501	NG	8.6	
	2	NG	Satt459	45311085	NG	23.8	
	18	NG	Satt288	51127425	NG	9.1	
Type: RIL P_1_: Misuzudaizu, *G. max*, Japan P_2_: **Moshidou Gong 503**, *G. max*, Japan	2	RAS2	Sat289b	47042650	6.3	10.6	Watanabe et al., 2004
	6	RAS1	Satt489-Satt100	23848501–31490622	14	26	
	20	RAS3	Satt367	2615587	3.0	6.1	
Type: RIL P_1_: Tokei 780, *G. max*, Japan P_2_: **Hidaka** 4, *G. soja*, Japan	6	qHS-C2	Sat_076-Satt307	16007694–46820673	5.9	18.5	Liu et al., 2007
	2	qHS-D1b	Satt459-Staga002	45311085–46281147	12.5	42.5	
Type: F_2_ P_1_: JS 71–05, *G. max*, India P_2_: **Brisa soya-1**, *G. max*, India	12	NG	Satt434	38428476	NG	3.9	Singh et al., 2008
	8	NG	Satt538	47395378	NG	4.5	
	6	NG	Satt281	6529270	NG	4.1	
	15	NG	Satt598	13653981	NG	4.2	
Type: F_2_ P_1_: PI 587982A, *G. max*, U.S.A. P_2_: **PI 594619**, *G. max*, U.S.A.	2	Isc	Satt459-Sat_202	45311085–45497649	88.1	65.6	Kebede et al., 2014
Type: RIL P_1_: Jidou 12, *G. max*, China P_2_: **ZDD03651**, *G. max*, China	2	qHS-2–1	Sat_069-Sat_183	43278769–44317044	2.51	5.54	Ai et al., 2018
	6	qHS-6–1	Sat_402-Satt460	16418553–44049891	6.64	12.94	
	9	qHS-9–1	Sat_399-Satt273	9095532–38799271	7.24	3.36	
	12	qHS-12–1	12_1-Satt353	18935–1687387	5.87	3.13	
	14	qHS-14–1	Satt577-Sat_287	675214–5287913	3.34	8.25	
Type: F_2_ and RIL P_1_: ZH 39, *G. max*, China P_1_: **NY27–38**, *G. soja*, China	2	NG	Satt274-Sat_198	45267040–45542863	13.3	17.2	Chen et al., 2019
	6	NG	6_0993–6_1068	18902966–20942203	13	17.8	
	3	NG	Sat_266-Sat_236	34016929–35595364	2.7	4.9	
	6	NG	Sat_402-Satt557	16418553–20218893	11.5	20.4	
Type: F_2_ P_1_: JS 335, *G. max*, India P_2_: **PI 424079 or PI 136620,** *G. soja*, India Type: RIL P_1_: DS 9712, *G. max*, India P_2_: **DC 2008–1**, *G. soja*, India	2	qScI-h 2–1	Satt703-Satt274	42580676–45267040	6.32	16.49	Chandra et al., 2020
	2	qScI-h 2–2	Satt274-Sat_202	45267040–45497649	11.83	26.66	
	5	qScI-h 5–1	Satt619-Satt545	35971621–36463025	3.42	5.96	
	12	qScI-h 12	Satt181-Satt434	36365787–38428476	3.85	6.22	
	13	qScI-h 13	Satt325-Satt252	13272992–16454986	3.01	6.71	
	16	qScI-h 16	Sct046-Satt686	2919448–13053666	3.72	6.74	

^#^Parent names in normal and bold fonts produce seeds with non-HS and HS trait, respectively. QTL, Quantitative trait loci; NG, the information was not given in the articles; LOD, logarithm of the odds; PVE, percentage of phenotypic variation.

## Genes related to HS in soybean

Although researchers have discovered a large number of QTLs related to soybean HS, it is still difficult to apply these QTLs to breeding due to the lack of accuracy and stability. Cloning causal genes underlying the HS trait of soybean and analyzing their regulatory mechanisms is of great significance for the utilization of HS trait. At present, three genes have been confirmed to regulate the formation of the HS trait in soybean seeds. Many studies have reported the presence of a common QTL on an overlapping region of soybean chromosome 2 (Keim et al., 1990; Watanabe et al., 2004; Liu et al., 2007; Zhang et al., 2008; Kebede et al., 2014). To understand the molecular basis of HS, Sun et al. (2015) developed two F_2_ populations by crossing the water permeable soybean cultivar Williams 82 and two *G. soja* accessions, and they delimited the QTL to a 22-Kb region containing two genes. Among them, a single nucleotide polymorphism (SNP; C to T at the 1,154^th^ site of its coding sequence) in *Glyma02g43700* (*Glyma.02g269500* in Wm82.v4), which could effectively distinguish between the parental genotypes and eight additional G. soja accessions, was found to be responsible for the occurrence of HS. *Glyma02g43700* encodes a calcineurin-like metallophosphoesterase transmembrane protein that is primarily expressed in the Malpighian layer of the seed coat, and the amino acid switch (Thr to Met at the 385th site of its protein sequence) resulting from the gene mutation was predicted to be located outside the membranes and to affect the protein structure, and thus increased the seed coat permeability (Sun et al., 2015). Jang et al. (2015) developed a near-isogenic line (NIL) of a permeable cultivar containing the HS allele qHS1 from wild soybean. Phenotypic analysis revealed that the qHS1 gene can increase the amount of β-1,4-glucans in the outer layer of palisade cells of the seed coat on the dorsal side of seeds, known to be a point of entrance for water (Ma et al., 2004; Meyer et al., 2007). Fine mapping and soybean transformation confirmed that an endo-1,4-β-glucanase gene, *Glyma02g43680* (*Glyma.02g269400* in Wm82.v4), was the causal HS gene. A point mutation (G to T at the 863^rd^ site of its coding sequence) introduced an amino acid substitution (Ser to Iso at the 288^th^ site of its protein sequence) in a substrate-binding cleft of the enzyme, possibly reducing or eliminating its affinity for substrates in permeable cultivars from various regions, while the allele from the HS genotype was found to promote the accumulation of β-1,4-glucan derivatives that reinforce the HS trait (Jang et al., 2015). The above two genes are both located within the previously reported chromosome 2 interval and are adjacent in position (Sakamoto et al., 2004; Liu et al., 2007; Kebede et al., 2014; Chen et al., 2019; Chandra et al., 2020) and may be related to the strength of the palisade layer of the seed coat. Wang et al. (2022) found that a polygalacturonase gene *PG031* (*Glyma.06G207300*) was specifically expressed in the flowers and reproductive tissues of soybean plants, and that it was strongly induced when the seed coat absorbs water. *PG031* underlies a QTL on chromosome 6 that can explain ~15% of phenotypic variation in seed coat permeability (Liu et al., 2007), and it has three haplotypes (289H, 289Y, Hap3) in the soybean population. Overexpression of the impermeable allele *PG031^289H^* in the permeable cultivar reduced the water permeability of transgenic seeds by reducing the level of pectins and decreasing intracellular spaces of the osteosclereid layer and parenchyma of the seed coat to decline water accessing the seed, and the 100-seed weight was also decreased. A genetic diversity analysis in a diverse soybean panel suggested that the permeable allele *PG031^289Y^*, associated with high water permeability and high seed weight, is experiencing ongoing artificial selection (Wang et al., 2022). In addition, two studies have predicted that several genes may be associated with HS of soybean based on comparative omics analysis. Whole-genome resequencing was conducted to identify the SNPs and InDels between genomes of cultivated and wild soybean in relation to their HS trait. Seven genes carrying nonsynonymous variants were found to play a probable role in influencing HS. Two of them (genes encoding type-I inositol polyphosphate-5-phosphatase-1 and E3 ubiquitin ligase) were found to segregate among the RILs in coherence with their water permeability scores and showed a preliminary association with the desirable water permeability characteristics (Ramakrishna et al., 2018). Recently, Wang et al. (2023) conducted transcriptomics and proteomics analysis of a hard-seeded chromosome segment substitution line R75 and its recurrent parent SN14, and multiple genes were found to associated with HS. Five of them were selected as candidates based on their expression patterns, functional annotations, SNPs, and protein structural changes. These genes may play important roles in the formation of the cell wall and seed coat, and showed significantly higher expression in seeds of R75 than in those of SN14. Kompetitive allele-specific PCR (KASP) markers were developed on the basis of their nonsynonymous SNP and showed high selection efficiency in distinguishing hard and nonhard soybean lines. The precise functions and regulatory mechanisms of these genes in regulating the HS trait require further research.

## Physical dormancy genes in other genera

In addition to those in soybean, genes in other plants have been found to regulate the level of seed coat permeability. In Arabidopsis, for example, mutants with altered proanthocyanidin production in the seed coat, such as *ban* (Albert et al., 1997) and *transparent testa 1*, *10*, and *13* (Debeaujon et al., 2000), and those defective in the secondary cell walls of the outer layers, such as *ap2* (Western et al., 2001), produce seeds that are more permeable than those of wild-type plants. Mutations of the laccase gene, *AtLAC15*, resulted in a reduction in lignin content and elevated permeability of Arabidopsis seed coats (Liang et al., 2006). An acyl-CoA:glycerol-3-phosphate acyltransferase gene, *AtGPAT5*, is required for the synthesis of suberin in the seed coat and root of Arabidopsis, and mutations in this gene result in a reduction in the very long chain dicarboxylic acids and ω-hydroxy fatty acids typical of suberin and a steep increase in seed coat permeability (Beisson et al., 2007). *Medicago truncata* is a model plant of the legume family due to its small genome, short growth period, and high genetic transformation efficiency, and it generally produces hard seeds. By screening of Tnt1 retrotransposon-tagged *Medicago truncata* lines, Chai et al. (2016) identified mutants that produce permeable seeds. A class II KNOTTED-like homeobox (KNOXII) gene, *KNOX4*, was found to be responsible for the loss of the HS trait in the seeds of the mutants. Mutation of *KNOX4* altered seed coat cuticle composition, and the knox4 mutant developed a structurally anomalous cuticle. Subsequent experiments showed that *CYP86A*, a gene associated with cutin biosynthesis, is a downstream target of KNOX4. Furthermore, the expression level of a seed coat-expressed β-ketoacyl-CoA synthase gene, *KCS12*, was found to be regulated by KNOX4. The concentration of C24:0 lipid polyester monomers are significantly decreased in *kcs12* mutant seeds, which exhibit irregular seed coats and lose the HS trait (Chai et al., 2021). These findings reveal a molecular mechanism by which KNOX4 and KCS12 control development of the seed coat and the HS trait. Common bean (*Phaseolus vulgaris*) is an important legume crop for human consumption, and some cultivars still retain a high level of the HS trait. To investigate the molecular basis of the rate of seed water absorption, an RIL population segregating for HS was constructed, and the *pectin acetylesterase 8* gene was found to be critical for the HS trait of common bean (Soltani et al., 2021). The nonfunction allele of *pectin acetylesterase 8* may be responsible for the development of permeable seeds, and was formed under strong selection pressure through domestication. In *Vigna stipulacea* Kuntze, another legume crop that inhabits mainly in South Asia, researchers have found that a cellulose synthase, a catalytic subunit gene *VsCESA7*, and a phospholipid sterol acyltransferase gene *VsPSAT1* may be responsible for the development of HS in their wild species (Takahashi et al., 2019; 2023). These studies show that the biosynthesis of some key chemical components is critical for seed coat permeability, and provide insights for investigation into soybean’s HS trait.

## Summary and future prospects

HS is a type of seed dormancy found in many angiosperms, which isolates the embryo from the external environment through its hard or impermeable seed coat, thus serving as an important mechanism for ensuring the survival and perpetuation of the species. This trait is particularly evident in leguminous plants, for example, wild soybeans, producing hard seeds. During domestication and breeding processes, most cultivated soybeans have lost the HS trait, which is beneficial for soybean production and processing. However, several cultivated soybean cultivars (e.g. HS cultivars in U.S.A., Japan and India, which has been used for QTL mapping of the HS trait, see **Table 1**) still exhibit the HS trait, and the expression of this trait is often influenced by environmental factors. Unveiling the physiological and molecular regulatory mechanisms governing the hard seed trait in soybeans is of significant importance for the utilization of wild soybeans in breeding, preservation of germplasm resources, and soybean production and processing. Previous studies on the HS trait in soybeans have made significant progress, and this paper provides a summary of this knowledge. The HS break of soybean seeds is mainly associated with microscopic cracks in the seed coat’s cuticle layer. The composition and content of fatty acids, phenols, and xylans may be related to the strength of the cuticle layer, thus participating in the formation of the HS trait. To date, nearly 30 QTLs related to the HS trait have been discovered on 13 chromosomes of soybeans, with significant differences in their contributions to phenotypic variation. These QTLs are very useful in molecular marker-assisted selection breeding in soybean and explaining the molecular mechanism underlying the HS trait. Among them, QTLs on chromosomes 2 and 6 have been repeatedly reported by different researchers, who have identified three causal genes responsible for HS based on this information. Through comparative omics analyses, some genes are also believed to be associated with the HS trait. In this article, we summarized the reported HS-related genes and their regulatory mechanisms in Arabidopsis and three legumes (*Medicago truncatula*, common bean, and *Vigna stipulacea* Kuntze), which mainly regulate HS by modulating the composition and content of the seed coat.

The above information provides insights into the mechanisms and utilization of the HS trait in soybeans, while there are still many unresolved issues in this research field. First, why do adverse or harsh environmental conditions induce the HS ratio in some soybean cultivars? Are there other regulatory mechanisms besides limiting the expansion of mature soybean cotyledons? For instance, do environmental factors affect the expression levels of certain genes in the seed coat, thus directly influencing seed coat composition that contributes to HS? Second, besides substances already reported by previous studies, what other key substances determine the strength and permeability of soybean seed coats? How is the synthesis of these substances regulated, and how do they affect seed coat structure? Studying HS and non-HS soybean seed coat caused by genetic or environmental factors through multi-omics techniques (e.g. transcriptome, proteomics, metabolomics) and analyze its regulatory mechanism through biochemical and molecular biology experiments may provide some clues to the aforementioned questions. Third, multiple HS QTLs have been discovered so far. Apart from chromosomes 2 and 6, which other genes on other chromosomes are involved in the formation of the HS trait in soybeans? Given that the HS trait is controlled by multiple genes, constructing genetically stable RIL or NIL populations by single seed descent or backcrossing method, respectively, will facilitate the cloning of other HS genes and the elucidation of regulatory mechanisms. Finally, how can currently cloned HS genes be utilized to breed soybean cultivars with moderately or fairly strong seed coats? Application of these cultivars can not only maintaining soybean production and food processing but also extending the storage time of soybean seeds, especially in humid regions. Modifying the expression levels and biochemical activities of HS regulatory genes/proteins through genetic engineering and gene editing techniques may be a preferable approach.

## Author contributions

YS: Funding acquisition, Investigation, Writing – original draft, Writing – review & editing. YG: Writing – original draft.
